# Supplementary material for: Controlling diverse robots by inferring Jacobian fields with deep networks
Source: Nature. 2025 Jun 25;643(8070):89–95. doi: 10.1038/s41586-025-09170-0 (PMC12221969; doi:10.1038/s41586-025-09170-0)
Supplement: Supplementary file 1 — More results, details and analyses, including discussions of trajectory tracking and alternative supervision strategies, further qualitative visualizations, details of robot systems and expanded related works. [file 41586_2025_9170_MOESM1_ESM.pdf]

nature portfolio

https://doi.org/10.1038/s41586-025-09170-0

Supplementary information

Controlling diverse robots by inferring Jacobian fields with deep networks

In the format provided by the authors and unedited

Controlling Diverse Robots by Inferring Jacobian Fields with Deep Networks

Supplementary Information

1 Dataset Collection

Details on Command Sampling. Training our dynamics model requires observations of varying robot configurations. Our Jacobian parameterization analysis in Extended Data Section 6 shows that, as a consequence of linearity and locality inductive biases of our parameterization, not all robot states that we wish to control at test time need to be observed at training time. For all results demonstrated in this work, random sampling of robot commands was sufficient. For the Allegro hand, we initialized the starting configuration of each trajectory in the dataset using a sparse grid of joint values; the subsequent commands in each trajectory are sampled randomly. A Bayesian exploration strategy is likely beneficial for systems with significantly higher degrees of freedom. We hope that our work can inspire future research endeavors in this direction.

2 Neural 3D Reconstruction and Neural Scene Representation

Limitations of pixelNeRF [61] in Occluded Regions. As a deterministic 3D reconstruction method, pixelNeRF will predict the mean of all possible 3D reconstructions conditioned on the observed image. In occluded regions of the robot, this can lead to blurry reconstructions. As evidenced by Extended Data Fig. 3 in our manuscript, our method retains the ability to control occluded regions effectively. Performance could likely be improved further by adopting a probabilistic method that can *sample* from all possible 3D reconstructions [52]. Further, conditioning the radiance and Jacobian fields on *global* information, such as that regressed by a potentially pre-trained vision transformer [8], would likely improve performance further.

Clarifications on the Jacobian Field Coordinate System. Our PixelNeRF is trained to make 3D predictions in camera coordinates relative to the input camera. This means that our model at deployment time does not rely on camera calibration and can be directly used in a different lab if the camera-to-robot transformation and scene appearances are within training distribution.

3 Alternative Supervision Strategies for 3D Jacobian Fields

While Differentiable Rendering is a convenient source of obtaining 3D supervision, it is not the only one. If high-quality depth cameras or motion capture are available, they can serve as the source of 3D information. We will discuss one such option that leverages RGB-D cameras in the following.

Supervising the Jacobian Field with RGB-D Cameras. Given multi-view RGB-D cameras, we may pair the 3D Jacobian Field with an alternative representation of 3D geometry that replaces the Neural Radiance Field. One choice are occupancy and signed distance fields [36]. These are functions that take a 3D coordinate as input and map them to the occupancy or the signed distance of that 3D point. 3D points obtained by unprojecting depth measurements from available RGB-D cameras can be used to directly supervise occupancy and signed distance fields [33], as they are assumed to be 3D surface points. Our 3D Jacobian Field can then be interpreted to lie on the surface encoded in the occupancy or signed distance field. To sample it, we may unproject a depth measurement from an RGB-D camera, find the closest surface point, and sample the Jacobian Field at that 3D position. The Jacobian Field can then be supervised via 2D optical flow in a manner identical to that described in the motion loss (Equation 10, manuscript).

Other architectures can be devised, such as a relying purely on point clouds obtained from RGB-D measurements as a 3D geometry representation and directly sampling the Jacobian field at such 3D points. However, such a

model cannot be used to predict or supervise the motion of *occluded* parts of the robot, which requires the sort of full 3D representation afforded by a radiance, occupancy, or signed distance field.

4 On the Value of 3D Representations

4.1 3D Enables Demonstration Transfer Between Viewpoints

Our 3D representation enables trajectory tracking of demonstration videos from viewpoints unavailable at inference-time (**Extended Data Fig. 4)**. Given a demonstration video recorded from an unavailable viewpoint, our PixelNeRF lifts each frame to 3D point clouds to form a high-dimensional trajectory (**Extended Data Fig. 4a**). Using a shape-based distance in 3D [14, 12], our model enables demonstration transfer from unavailable viewpoints. We did not cherry-pick designs for the model predictive controller and used the same control algorithm regardless of whether the reference trajectory is 2D or 3D.

Experimental Results. We test demonstration transfer on the Allegro hand, whose analytical model is reliable for performance analysis. Our framework enables the Allegro hand to track demonstration videos specified in unavailable viewpoints. For every demonstration trajectory, we run our method ten times to compute the final error's statistical average and standard deviation, measured as the difference between the final achieved joint angles and the ground truth joint angles in degree. We find that our method achieves a high-quality median error of 2.2 degrees, as reported in **Extended Data Fig. 4c**.

4.2 3D Eliminates Motion Ambiguity Between Viewpoints

A key challenge of learning robot control from 2D motion alone is that 2D observations are inherently *ambiguous* in the sense that many potential 3D motions map to identical or almost identical 2D motion. This form of motion ambiguity is a well-studied problem in computer vision [51, 6]. For instance, observing the HSA platform directly from the top will lead to vanishing optical flows for extending and shortening of the platform. For multi-fingered robots, bending or moving diagonally induces almost identical optical flows when observing the robot hand from the side.

Our novel view synthesis objective forces our network to learn a single 3D Jacobian field that correctly predicts motion in *all* available camera perspectives, resolving the ambiguity.

5 Details on Trajectory Tracking

The algorithms described in this section can be found at `github.com/sizhe-li/neural-jacobian-field/`.

5.1 Model Predictive Control Algorithm

Our model predictive control (MPC) algorithm presented in Algorithm 1 is general for both the 2D and 3D use cases. The only differences are the ways in which the current state encoding is computed and the choice of the cost function. We describe the MPC algorithm below and will present the case-by-case details in separate paragraphs.

5.2 2D Trajectory Tracking

Demonstration Preprocessing Details. Given a demonstration video, we use an optical flow mask and a segmentation mask predicted by a foundation model [21] to initialize tracking points to be inside the robot. We use the initial points to convert the video to point tracks using TAPIR [13]. To create 2D waypoints, we refer the readers to the last paragraph of this section on tracking target details. We create multiple tracking targets empirically by storing a waypoint in the demonstration video every five frames. A waypoint contains the pixel locations of all points and the TAPIR features of all points. TAPIR features are later used during model predictive control to initialize the corresponding key points in the current observation at the beginning of a waypoint segment.

State Encoding Details. At deployment, we convert the current RGB observation to point tracks. At the zeroth step of each waypoint, we use the stored TAPIR features to find corresponding points in the current image [13]. For all other steps, we run TAPIR forward to predict the current key point locations.

Algorithm 1 Model-Predictive Trajectory Tracking

Inputs:
  Reference trajectory (2D or 3D) $\tau_{1:T} = (\tau_1, \tau_2, ..., \tau_T)$
  Threshold to next goal ϵ
  Cost function (2D or 3D) $\mathcal{L}(\cdot, \cdot)$
Initialize:
  $t \leftarrow 0$
while $t < T$ **do**
  $X_{curr} \leftarrow$ encode current state from RGB ▷ 2D or 3D, details in Sec. 5
  $loss \leftarrow \mathcal{L}(X_{curr}, \tau_t)$
  while $loss > \epsilon$ **do**
    $X_{curr} \leftarrow$ encode current state from RGB ▷ 2D or 3D, details in Sec. 5
    $\delta u \leftarrow \arg\min_{\delta u} \mathcal{L}(J(X_{curr})\delta u + X_{curr}, \tau_t)$ ▷ Details in Sec. 5
    Execute command δu
    $loss \leftarrow \mathcal{L}(X_{curr}, \tau_t)$
  $t \leftarrow t + 1$

Command Optimization Details. In the 2D case, we use the Jacobian Fields to find the best robot command that takes the current point locations closer to the location of the next waypoint. Consider a pixel location (i, j) and its current target (i', j'). Given a command δu, we can compute the current 2D optical flow at (i, j) by volume rendering the 3D advections of 3D sample points along that ray, following Equation 7 in the manuscript. By advecting 2D pixel locations, we obtain the predicted location (i^+, j^+) via our dynamics model. We use L2 distance and solve a gradient descent program that optimizes the command to minimize the distance between the prediction (i^+, j^+) and the target (i', j'). We leverage TAPIR visibility predictions and zero out distance loss on point tracks that are invisible in the current frame.

5.3 3D Trajectory Tracking

Demonstration Preprocessing Details. We lift videos to 3D states using our neural 3D reconstruction module, PixelNeRF. We sample 100x100x100 points inside a specified 3D grid. For every point, we query the density field and store its 3D spatial location if the density is larger than 20 at that spatial location. We convert each RGB frame to a point cloud by repeating this procedure on every frame. We form a demonstration trajectory by storing the point cloud locations of each waypoint frame.

State Encoding Details. We apply the same procedure for demonstration preprocessing to encode the current image observation to a 3D state. For an RGB observation, we use rejection sampling to obtain high-density points inside the same specified 3D grid.

Command Optimization Details. We use Wasserstein-1 distance to measure the shape differences between two point clouds in 3D [12, 14]. We clarify that Wasserstein distance can accept two point clouds with different numbers of points, as it measures the transport potentials. We lift the current RGB observation to a 3D point cloud $X \in \mathbb{R}^{N\times 3}$. For every current 3D point $x \in \mathbb{R}^3$, we query their Jacobian Fields quantity $J(x|I)$. Given a robot command δu, we use the flow field to advect the current point cloud and obtain X^+ via our dynamics model. We use Wasserstein-1 distance to solve a gradient descent program that finds the best command to transform the current point cloud closer to the tracking target.

6 Details on Jacobian Parameterization Ablations

Next, we show analysis and experimental results on how the Jacobian parametrization of scene flow enables out-of-distribution generalization to unseen motion at test time, as well as improved sample efficiency. We first observe that mechanical systems made from continuum solids display *linearity* and *locality* [5, 22, 30], providing formal justification for the empirically demonstrated generalization of our system.

Observation 1: Linearity from Local Theory of Smoothing [35] We model *differential* kinematics by linearizing the system dynamics, which represents 3D motion fields induced by *small* control commands δu. In this regime, it is well-known that the 3D motion δx of robot 3D points can be described by the space Jacobian *and is thus a linear function of control commands*, i.e. $\alpha\delta x = \frac{\partial f}{\partial u}(\alpha\delta u)$. This is powerful, as completely specifying the system dynamics for a particular configuration u' requires only $n \times 3$ linearly independent observations of pairs of

control commands and induced scene flow, as this fully constrains the space Jacobian for a given configuration for a system with n control channels. For instance, one need not observe the motions for *both* $-\delta u$ and δu; it suffices to observe *one* of them. Similarly, one need not observe δu and a scalar multiple $\alpha \delta u$; again, one of them in the training set suffices. This is in stark contrast to parameterizing f as a neural network that directly predicts scene flow given an image and a robot command since the neural network does *not* model these symmetries and will thus require orders of magnitude more motion observations to adequately model the system dynamics.

Observation 2: Spatial Locality of Mechanical Systems Robot commands often result in highly localized spatial motion. Here is an intuitive example:

- **Locality of independent kinematic chains [30].** Consider a multi-fingered robot hand (**Fig. 2**), commanding the thumb motors in this configuration leaves the little finger still. The manipulator Jacobian field as a physical quantity is locally smooth across space.

Observation 3: Spatial Compositionality of Mechanical Systems Robot commands often result in spatial motions composed by the influences of individual command channels. Here is an intuitive example:

- **Compositional kinematic joints [30][35].** Consider the simple two-joint robot finger in **Fig. 2**, for a material point inside the second segment (as a solid domain [5]), its motion is computed as the integral over individual command channels in the Jacobian tensor field. In the example of **Fig. 2**, the 2D motion is the summation over two Jacobian channels.

6.1 Experimental Evidence of Benefits of Locality, Linearity, and Compositionality

We now provide experimental evidence across a diverse set of real-world and simulated domains to show that the inductive bias of our Jacobian parameterization leads to better predictions and out-of-distribution performance.

Real-world Experiments with Allegro Hand. We benchmark our Jacobian field parameterization with a baseline where we directly predict the scene flow δx conditioned on a robot command δu. Specifically, we provide an MLP with the per-pixel feature vector as well as the motor command and train it to output the 3D scene flow on the same training data and with the same CNN backbone as our Jacobian Field. We compare this baseline against our approach both quantitatively and qualitatively for the flow prediction task, reported in Extended Data Tab. 3 and Extended Data Fig. 5.

The highly dexterous Allegro hand has 16 degrees of freedom, with independent kinematic chains (i.e., four fingers). The dexterity of the system creates combinatorial complexity for modeling the configurations of the robot hand. In Extended Data Fig. 5, we show that the baseline model fails to predict the correct motions for the robot hand on the validation dataset, where our method succeeds. We note that the baseline method fits the training set well, suggesting that the problem is generalization. Quantitatively, as shown in Extended Data Tab. 3, our model attains significantly lower flow prediction error.

Simulated Experiments. We created a physics-based simulation of two conventional robotic systems [30][35] in 2D. We create our model and the baseline model in the same fashion as our real-world comparison above. Our model predicts a 2D Jacobian at a pixel conditioned on an image observation, whereas the baseline model directly predicts the 2D flow at a pixel conditioned on the image observation and the robot command.

1. **(Pusher Environment [11]; Fig. 1)** The environment contains a spherical robotic pusher [11]. The robot can move freely in 2D space and is steered by a 2D velocity command $\delta u \triangleq (x, y)$, where $x, y \in \mathbb{R}$.
2. **(Dexterous Finger Environment [35]; Fig. 2)** The environment contains a 2 degrees-of-freedom robot finger. The robot finger is commanded by a 2D joint velocity command $\delta u \triangleq (u_1, u_2)$, where $u_1, u_2 \in \mathbb{R}$ control the rotations of each motor respectively.

Results of Pusher Experiment (Fig. 1 Tab. 1). Training on just two trajectories of the robot moving down and moving right. We investigate whether the learned dynamics model generalizes to unseen spatial locations and unseen robot command magnitudes and directions.

We find that the Jacobian model can generalize to the whole space of $\mathbb{R}^2$ configurations, and the whole space of $\mathbb{R}^2$ motion magnitudes and directions. This is substantiated by **Fig. 1 and Tab. 1**, where we task the robot to draw curves and move in directions at locations unseen during training. In comparison, the direct flow prediction model fails to genearlize to unseen motion magnitudes and directions, and thus is unable to control the robot.

Results of Finger Experiment; (Fig. 2; Tab. 1). For training, we create two trajectories that represent just

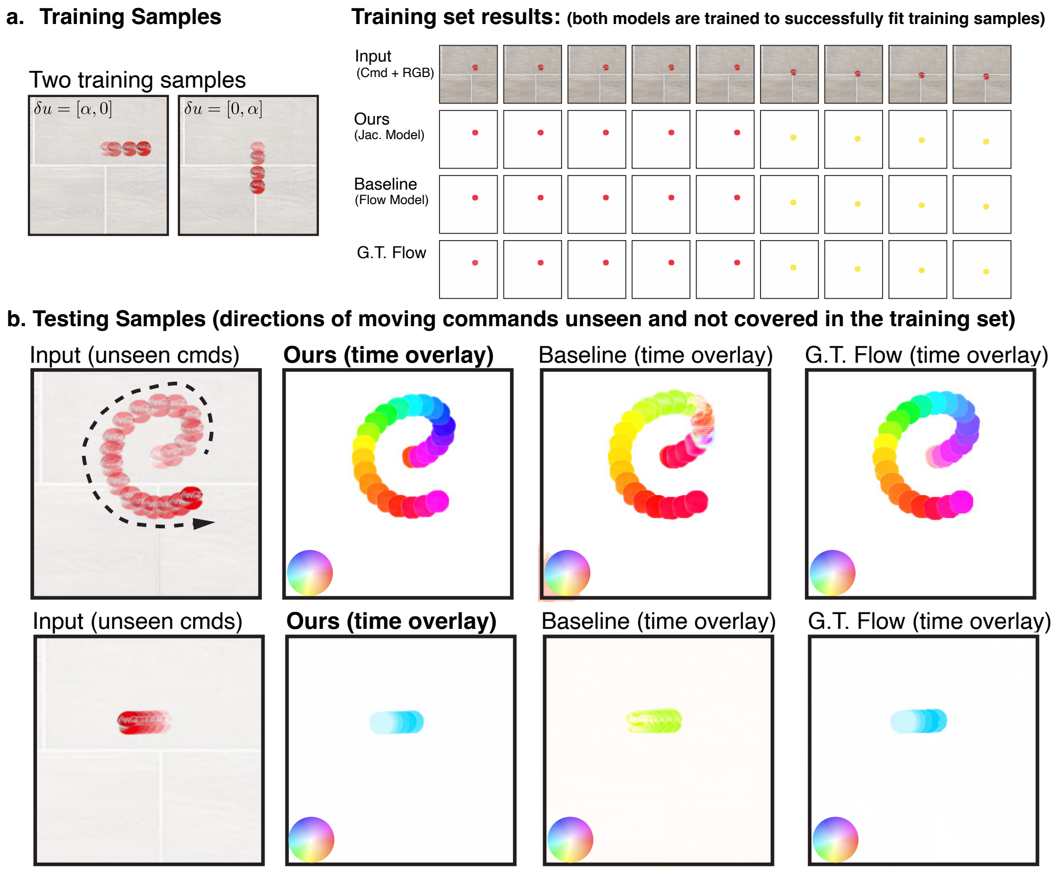

Figure 1: **Evaluations of Jacobian properties using 2D Pusher Environment. a,** Training on just two trajectories of the robot moving down and moving right, both our model and the direct neural flow baseline are able to fit the dataset well. **b,** Our Jacobian model is able to generalize to unseen spatial locations and unseen robot commands. Our model predicts correct optical flow values, as shown by the time overlay. In comparison, the baseline model fails to generalize due to the lack of inductive biases.

two types of commands: rotating the first motor only, rotating the second motor only. For testing, we turn the first motor to an unseen configuration and rotate the second motor and vice versa. We ensured that the baseline model was not underfitting. As observed in **Fig. 2**, the baseline model can perfectly reconstruct training samples.

We find that our model generalizes to the out-of-distribution test scene, solving both the forward and the inverse problems. In comparison, we found that the baseline model fails at unseen configurations, incorrectly predicting motions not only on surfaces of the second finger segment but the first one as well, i.e. not successfully disentangling parts of the kinematic chain.

Experimental evidence above, from both the real world and the simulated domain, substantiates our claim – our Jacobian parameterization, through capturing spatial locality and spatial symmetry of the robotic system, dramatically improves out-of-distribution motion prediction.

	Pusher Environment			Finger Environment
Optical flow errors (pix.)	Circle	Moving Left	Moving Up	Closing Second Joint
Direct Flow Model	0.635	0.0985	0.0969	8.651
**Jacobian Model (Ours)**	**0.0247**	**0.00173**	**0.00184**	**0.178**

Table 1: **Simulated comparison between our Jacobian model and the direct neural flow baseline.** The mean errors are computed by evaluating each trajectory 10 times. We find that our model outperforms the baseline model. Our model is able to generalize to unseen evaluation samples. We highlight that this baseline model is modeled after the architecture described by Xu et al., 2020 [60].

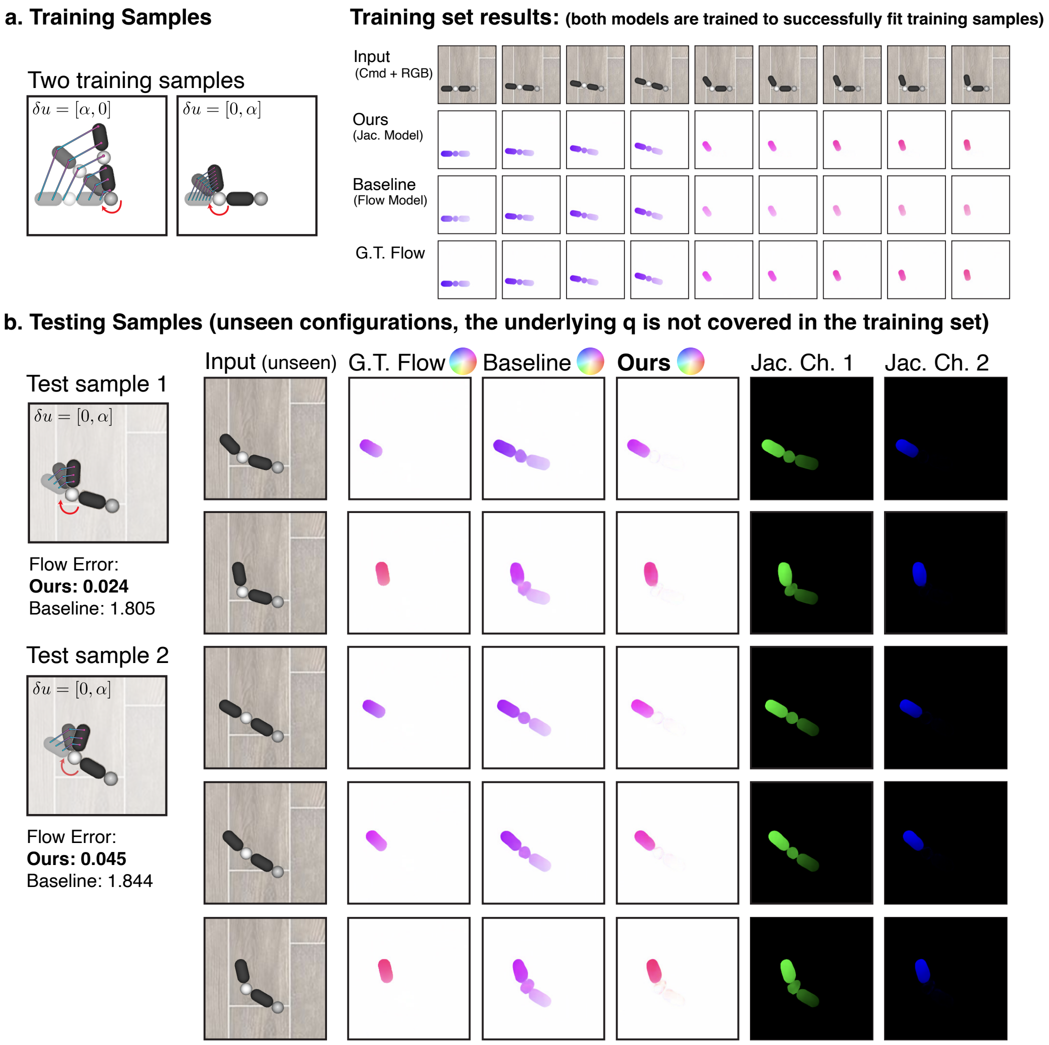

Figure 2: **Evaluations of Jacobian properties using 2D Finger Environment. a,** Training on just two trajectories of the robot rotating the first and the second motors, both our model and the direct neural flow baseline can fit the dataset well. **b,** Our Jacobian model can generalize to unseen finger configurations and robot commands. Our model predicts correct optical flow values. In comparison, the baseline model fails to generalize due to the lack of inductive biases.

7 Details on Neural Rendering Ablations

We ablate the neural rendering part of the Jacobian fields by assuming that the model is given depth as input. Keeping the rest of the architecture unchanged, we train a pixel-aligned 3D Jacobian Fields model. This model is supervised using the same losses on depth and flow. Specifically, it needs to predict the correct 3D Jacobian at each pixel so that the resulting 3D scene flow, when projected to 2D, matches the observed optical flow. We report quantitative results in **Table 2**, and provide qualitiative results in **Figure 3**. We find that this field-free 3D Jacobian baseline performs *significantly worse*. Specifically, the baseline regularly fails to disentangle the sensitivities of different surface points to different actuators, predicting incorrect Jacobians. We hypothesize that this is due to a lack of multi-view supervision: Without a NeRF that allows us to easily check for which parts of the robot are occluded in which view at training time, supervision for the 3D scene flow stems only from the same camera perspective that is fed to the image encoder.

Optical flow error (pix.)	**Mean**	**Std**
Jacobian Field + Depth Input	1.161	1.650
**Jacobian Field + Neural Rendering**	**0.155**	**0.053**

Table 2: **Ablation on neural rendering on the pneumatic hand.** We removed the neural rendering part of Jacobian Fields by assuming that the model is given depth as input. Keeping the rest of the architecture unchanged, we train a pixel-aligned 3D Jacobian model. The model performs significantly worse numerically. Qualitatively, we found that this model fails to disentangle the sensitivities of surface points to different actuators, predicting incorrect Jacobians that over-explain the data. We hypothesize that this is due to a lack of multi-view supervision.

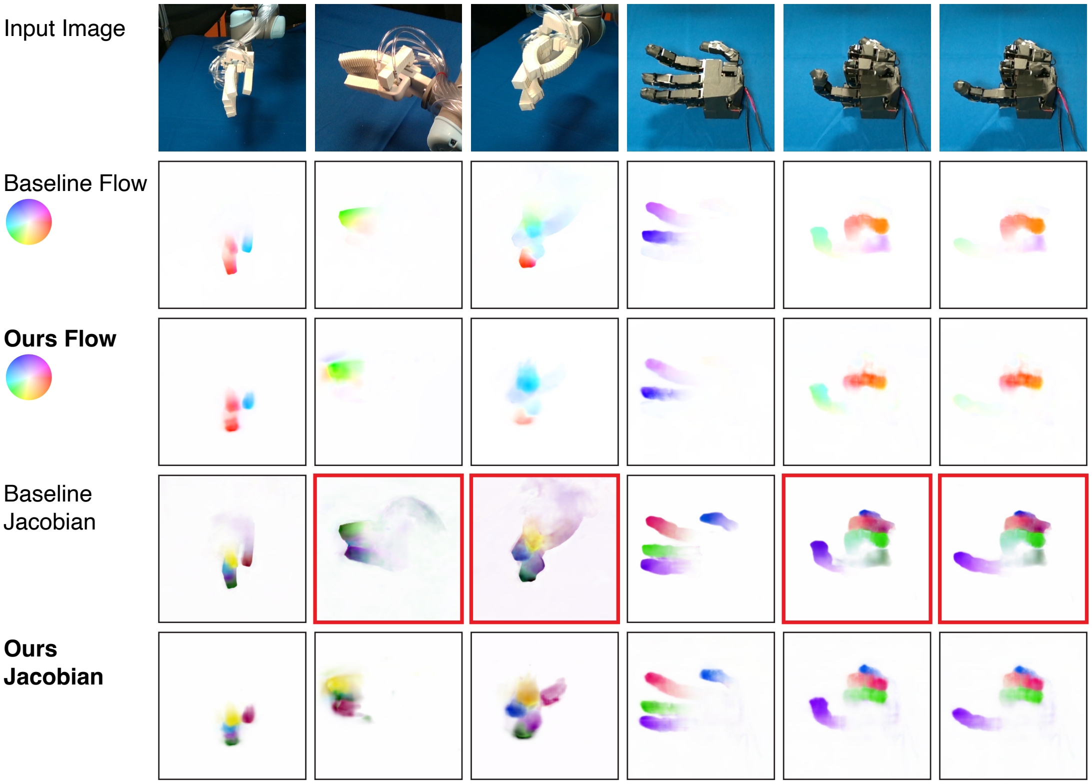

Figure 3: **Ablation on neural rendering on the Allegro Hand.** We removed the neural rendering part of Jacobian Fields by assuming that the model is given depth as input. Keeping the rest of the architecture unchanged, we train a pixel-aligned 3D Jacobian model. The model performs significantly worse numerically. Qualitatively, we found that this model fails to disentangle the sensitivities of surface points to different actuators, predicting incorrect Jacobians (**red boxes**) that over-explain viewpoints with occlusion. We hypothesize that this is due to a lack of multi-view supervision. For each robot, we show one successful example from both models, followed by two examples highlighting the baseline's failure modes.

8 Details on Robustness Analysis of Neural 3D Reconstruction

8.1 Robustness Against Visual Occlusions

We apply domain randomization techniques [53, 37, 15] to the input images during training. This process trains our framework to be robust against occlusion, background changes, and other visual distribution shifts. For every input frame collected in our video dataset, we apply a motion threshold to the point tracks and use the Segment Anything framework [21] to obtain a binary mask corresponding to the robot. We apply background domain randomization as described and implemented in [15]. We apply foreground domain randomization by overlaying natural images randomly sampled from the coco dataset [27] on our training video frames. Before overlaying, we randomly crop the coco images and resize them to be smaller than the dataset video frame size. We highlight that the target images used to create supervision are not augmented, and are the undisturbed original images, depths, and optical flows.

This trains the neural single-image-to-3D module to be robust against background changes and partial occlusion. As empirically observed in Extended Data Fig. 3 and quantitatively tested in Fig. 4, our framework's depth and Jacobian predictions are robust against visual perturbations.

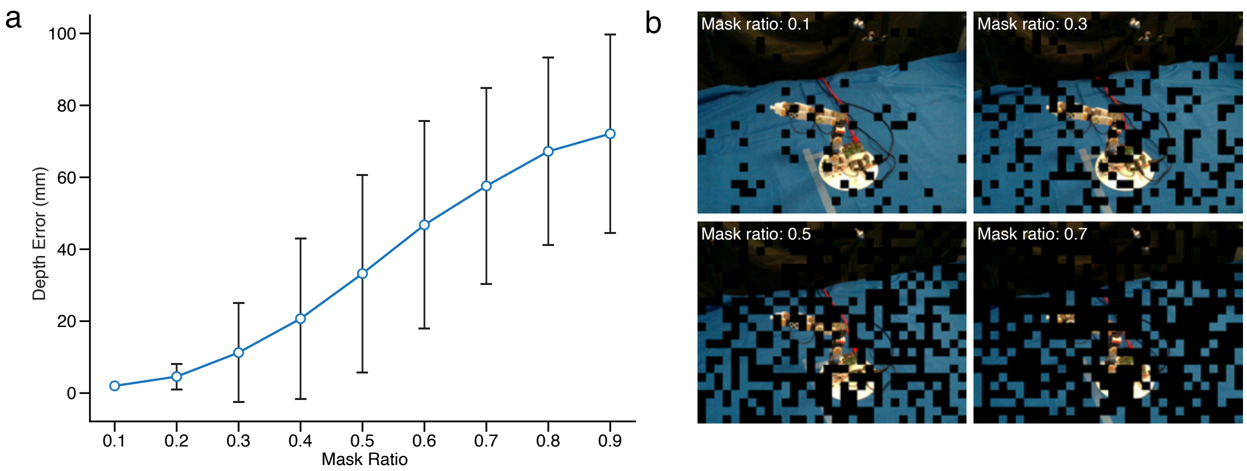

Figure 4: **Sensitivity analysis of depth prediction. a,** We evaluate the depth reconstruction quality under out-of-distribution scenarios. Given a collection of testing images, we perturb these images with black patches sampled at random locations that achieve increasingly larger mask ratios. We plot the mean depth error over the mask ratio and visualize the standard deviation as the error bar. **b,** Overlaying black patches on an example input image with increasingly larger mask ratios.

8.2 Robustness Against Scene Perturbations

We test our approach's ability to identify and control the Allegro hand under heavy appearance perturbations. Recall from Section 5.5 in the main paper that, during training, our data augmentation process samples background noise to make the model invariant to background perturbations.

During testing, we introduced significant perturbation to the scene's geometry and appearance by fencing off the Allegro hand with several pieces of white cardboard, shown in Fig. 5a. We used the 3D trajectory tracking scheme described above to control the Allegro hand to close. We report the quantitative and qualitative results in Figure 5. We find that our method is able to control the Allegro hand to follow 3D trajectories in the face of scene perturbations. We numerically find that our approach can obtain high-quality tracking results, achieving a median joint error of 2.89 degrees.

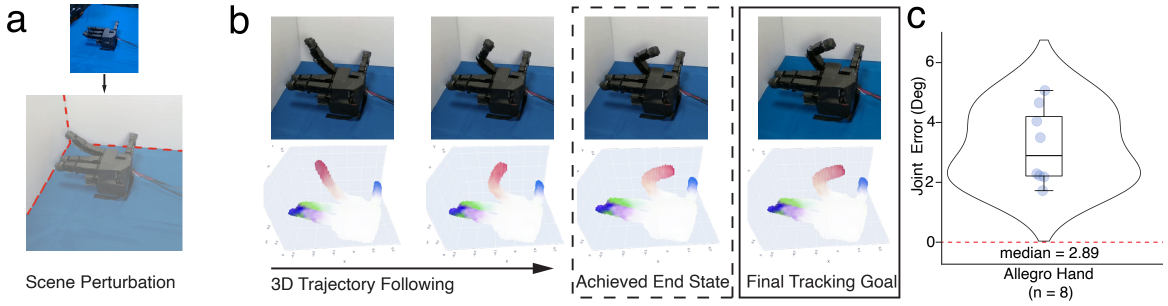

Figure 5: **Additional evaluations on scene perturbation. a,** We conduct additional evaluations on the robustness of our method against scene geometry and appearance perturbations. We placed several pieces of cardboard to fence off the hand, perturbing the visual scene. We run the reference trajectory eight times **b,** We visualize the predicted 3D state of our method as it controls the Allegro Hand to follow a 3D trajectory. **c,** We run each trajectory eight times to compute the mean and standard deviation of the errors, measured as the joint angle differences between the achieved end state and the final tracking goal.

Open challenges While our proposed neural 3D reconstruction module can leverage large visual foundation models, many settings remain challenging and still represent the holy grail for many efforts across robotics, computer vision, and machine learning. We hope that our model can be scaled and improved for the following outstanding Challenges

1. Training Jacobian Fields on a robot in Lab A, generalizing to the same robot placed in a different Lab B.

2. Relocating the camera to an arbitrary viewpoint in the scene and generalizing to that view.
3. Changing the robot's morphology after the model is trained. E.g., uninstalling a finger from the Allegro hand and installing the Allegro hand to an arbitrary robot arm.
4. Generalization challenge due to the combinatorial complexity of the configuration space. Mounting a robotic hand onto an arm, the motion of any surface on the robot hand is now subject to the arm's configuration and motion.

9 Details on Comparisons with Analytical Dynamics Model

We conducted experiments comparing our learned Jacobain model against analytical models for the Allegro hand and the HSA platform.

9.1 Allegro Hand

Using Drake [50], a state-of-the-art rigid robot simulation, we quantitatively found that our model performs well in predicting the angles of the Jacobian vector for each command channel, resulting in an average error of 7 degrees. Qualitatively, we visualize our comparison against the analytical model in Extended Data Fig. 6.

Experimental Procedure. We sample 3D points inside the collision volume of the Allegro hand in Drake. We analytically compute the transformed Jacobian at the queried point by extending the kinematic tree. For our Jacobian field, we similar sample 3D points inside the scene uniformly using a 3D grid. Using Procrustes analysis [23], we compute the 3D transformation that registers the analytical Jacobian point cloud to our predicted Jacobian point cloud. For every point in the predicted Jacobian field, we find the five nearest neighboring points from the analytical Jacobian field and take their average Jacobian matrix. We compare the angle differences of each command-channel vector inside between the two Jacobian matrices.

9.2 HSA Platform

Using the analytical model of the HSA platform proposed in [48], we compare our learned model by using an expert-designed model as the oracle. The analytical model of the HSA platform from [48] is a 2D model that only supports 2D motion. Notably, it does not account for motions that are inherently 3D, such as the twisting motion that the HSA platform is capable of. The model uses a discrete Cosserat approach from [39] and assumes that the backbone of each HSA actuator maintains a constant strain. To make it amenable to our formulation, we adopt a quasi-static approximation to map motor actuator commands to their corresponding state variables. However, this model struggles to capture the dynamics accurately in straight configurations due to a singularity encountered when the HSA actuators are straight, as the curvature of the HSA backbone becomes zero. Therefore, we evaluate their model in different 2D configurations where the HSA is bent.

We emphasize that the expert-designed model is not a perfect reflection of the physical reality of the HSA system. It uses simplified 2D geometry and does not model or support 3D twisting motions. To the best of our knowledge, our model is the only current solution for modeling the 3D twisting and tilting dynamics of the HSA platforms. The current best expert-designed models can only support 2D tilting motions.

Experimental Procedure. Since the analytical model only supports 2D HSA configurations, where the HSA legs on the two sides are symmetrical, we command the HSA platform to compliant testing configurations. For our Jacobian field, we sample 3D points inside the scene to obtain the predicted Jacobian point cloud. We project the 3D point cloud to 2D and use Procrustes analysis [23] to align with the coordinate system of the 2D analytical model. For every point in the predicted Jacobian field, we find the five nearest neighboring points from the analytical Jacobian field and take their average Jacobian matrix. We compare the angle differences of each command-channel vector inside between the two Jacobian matrices.

10 Details on Robot Systems

10.1 Pneumatic Hand

Based on a design first introduced in [32], the pneumatically actuated soft robot hand is 3D printed in one piece using vision-controlled jetting [7]. This printing technique is based on inkjet deposition and enables the combination of soft and rigid materials. The fingers are based on PneuNet actuators [20], and the palm features a

rigid core surrounded by soft elastomer skin. The hand does not require manual assembly after printing, and the total fabrication cost is around $300. The hand is driven by a 15-channel proportional valve terminal (MPA-FB-VI, Festo). Each channel can be individually controlled to adjust the pressure in each of the 15 degrees of freedom (DoF) of the hand. For our experiments, we employ two versions of this hand. One is marked by a pen with blue crosses and operated standing upright in the workspace. The other is unmarked and mounted to the tool flange of an industrial robot arm (UR5, Universal Robots A/S). We use the two shoulder joints of the arm to move the hand horizontally and vertically in the workspace.

10.2 Allegro Hand

The Allegro hand (Wonik Robotics Co. Ltd.) is a commercially available, 16-DoF anthropomorphic robot hand with four fingers [1]. Each of the fingers is actuated by four servos and has soft silicone padding at the fingertips. The servos provide joint position feedback and can be torque-controlled via current control. The Allegro hand is popular among researchers across academia and industry, but also expensive, with a price tag upwards of $15,000 [24, 43, 16, 64].

10.3 HSA Platform

The HSA platform is a servo-driven, 4-DoF soft robotic platform based on compliant actuators made out of handed shearing auxetics (HSAs). Introduced in [28], HSAs are metamaterials with patterns that couple extension with shearing. Tiled on the surface of a cylinder, HSAs enable linear actuators that extend when twisted. These actuators are 3D printed from polyurethane resins (FPU 50, Carbon Inc.) via digital light projection, according to [55]. Four actuators are arranged in a 2×2 configuration, joined rigidly together at the top, and each driven by a servomotor at the bottom. The resulting robotic platform has three rotational DoFs that mimic the movements of a human wrist and one translational DoF that allows it to extend and contract. The HSA platform costs about $1,200 to make, with the four servos (Dynamixel MX-28, ROBOTIS) at $260 each dominating the cost. Even though a model that relates servo position feedback to platform pose can be learned, the relationship breaks down when the compliant HSA platform deforms in response to external forces [63]. HSA-based robots can be sensorized via fluidic innervation [56] and embedding internal cameras [62]. However, each of these methods comes at the price of significantly increased fabrication complexity.

10.4 Poppy Ergo Jr

The Ergo Jr is an ultra-low-cost 6-DoF robot arm for educational purposes and part of the DIY open-source robotics platform called the *Poppy Project* [3, 25]. The Ergo Jr is driven by six low-cost servos (Dynamixel XL-320, ROBOTIS) and can be assembled in 30-45 minutes [2]. Apart from the servos and the electronics, its parts are 3D printed on a consumer-grade desktop FDM printer (P1S, Bambu Lab). The Ergo Jr cost us $270 in total (Extended Data Tab. 3), which can be reduced further if the servo control board was custom-made instead of bought off-the-shelf. The Ergo Jr is not equipped with any sensors other than the servo encoder. Past research [41, 10, 17] describes this robot as difficult to model due to its low manufacturing quality and significant backlash in its kinematic chain.

Part	Quantity	Unit Cost	Total
ROBOTIS Dyanmixel XL-320 servos	6	$ 26.90	$ 161.40
Pixl Poppy Ergo Jr servo control board	1	$ 32.47	$ 32.47
ROBOTIS RS-10 rivets	1	$ 6.60	$ 6.60
ROBOTIS rivet tool	1	$ 1.10	$ 1.10
Raspberi Pi 3 Model B+ board	1	$ 48.99	$ 48.99
Gigastone 8GB microSD card	1	$ 3.30	$ 3.30
PwrON 7.5V 2A AC DC power supply	1	$ 9.99	$ 9.99
Vabogu-CAT8 ethernet cable	1	$ 3.99	$ 3.99
Bambu Lab PLA filament for 3D printed parts	108 g	$ 20 per kg	$ 2.16
Screws, nuts, standoffs (estimate)			$ 2.00
**Grand Total**			**$ 272.00**

Table 3: Bill of materials for Poppy Ergo Jr arm.

11 Related Works

Analytical Dynamics Models. Conventionally, experts model robotic systems on a case-by-case basis. Experts design a state vector $\mathbf{q}$ and embed sensors that fully describe the robot's morphology. This approach has proven successful in modeling rigid robotic platforms, where joint angles and velocities are the state variables fully describing the current geometry of the robot. After modeling the robots, analytical simulations are used to plan motions and control the robot to perform physical tasks [30, 50][35]. However, the conventional modeling strategy is challenging to apply for soft and hybrid soft-rigid systems. Designing the state vector and embedding sensors that measure the continuous state of a deformable system are two challenging tasks.

From a mechanical perspective, state vectors have two primary purposes: discretizing the (1) **geometry** of a system and (2) **time** for evolving differential equations. Achieving (1) requires the ability to spatially represent the geometric configuration of a system. In contrast to rigidly jointed segments whose spatial configuration can be reduced to joint angles, deformable solids require continuous spatial parameterization of the physical fields acting on the solid domain. Standard choices are Lagrangian particles or meshes and Eulerian grids [5, 47, 22]. Our work spatially discretizes the dynamics by representing the geometric configuration of a system as density fields, learned purely from reconstructing image observations observed from different viewpoints [34, 61]. Achieving (2) requires specifications of constitutive relationships, such as stress-strain laws that push forward temporal transients of continuum solids [5, 47]. Material energy functions are written by experts to approximate these laws. In contrast, our approach achieves this goal by relaxing and linearizing the system's dynamics to transitions between steady states [35]. Learning Jacobian Fields amounts to grounding visual features to physical features of a system, which implicitly describes material functions acting on solid domains. For example, our Jacobian Fields linearizes the stress-strain behaviors of handed shearing auxetics [28], as illustrated in Fig. 2e in our manuscript.

Sensing in soft and bio-inspired robotics faces several critical challenges due to the deformable and nonlinear nature of soft materials. Unlike rigid robots, where sensors are attached to predictable structures, soft robots undergo continuous shape changes, resulting in complex and high-dimensional data that are difficult to interpret [40, 54]. Embedding soft sensors is particularly challenging because the sensors must be stretchable, durable, and capable of withstanding large deformations without compromising the robot's mechanical properties [58]. Additionally, they must maintain consistent signal quality despite being integrated into highly elastic materials, which often leads to signal drift or noise over time [46]. Another difficulty is preserving the robot's softness and flexibility since integrating rigid components or bulky electronics can hinder the robot's performance and adaptability. Recent efforts focus on integrating novel materials and leveraging advanced modeling techniques to address these limitations [18]. These challenges make soft robot sensing a multidisciplinary effort requiring expertise in robotics, materials engineering, and design [31]. Our work is well-positioned to address these challenges, as our framework unshackles the hardware design of robots from our ability to manually model them, which in the past has dictated precision manufacturing, costly materials, extensive sensing capabilities, and reliance on conventional rigid building blocks.

Robot Kinematics and Dynamics Learning. Recent advancements in machine learning and spatial computing have opened research efforts on learning dynamics of physical systems from observing state transitions. [4, 42, 38]. We focus our discussion on the scope of robot dynamics [26, 60, 45]. First, past works have focused on a different problem class. To the best of our knowledge, no prior works have investigated how to learn to visually perceive, represent, and control robotic systems with *imprecise or unavailable sensors, unknown morphology, and unknown dynamics*.

The majority of past works in robot dynamics modeling do not study representing the robots themselves. Refs. [26, 45, 60, 44] focus on how pushing actions by a gripper end-effector will change the shape of a deformable body or will move a rigid body on the table. The inputs to these methods are typically the locations and directions for the pushing actions. Consequently, these methods can only be used for robots that follow the same kinematic, dynamic, and functional requirements. Robots also need to come with expert-written robot modeling software to compute control commands since the inputs to these methods are typically end-effector changes. Additionally, these assumptions make it difficult for these methods to leverage pre-trained large visual foundation models [19, 49] that our work can take advantage of, as they take pre-processed perceptual inputs (e.g., 3D object bounding box, segmented object point clouds) rather than the whole visual observation. Our closest work might be Ref. [9], which explores learning the unknown morphology of the robot using implicit representations. Our work is different in the capability and the problem class. In particular, Ref. [9] does not study how to *perceive* robotic systems from vision. It fits a neural mapping from joint angles, measured from precise embedded sensors to a signed distance

field. To control a rigid robot arm, Ref. [9] relies on human-in-the-loop attachments of 3D motion capture markers to the end-effector to learn a second neural mapping. Given that embedded sensors are unavailable in soft and bio-inspired robots and that attaching motion capture markers is not scalable for deformable robots, Ref. [9] and prior works cannot directly model the robotic systems described in this work.

In addition to the difference in problem class, our ablative experiments demonstrate the values of our proposed Jacobian parameterizations enjoy the advantages of linearity and locality, which enable sample efficiency and out-of-distribution (OOD) generalization to unseen inputs. In contrast with the direct neural flow prediction baseline, our Jacobian parameterization improves sample efficiency and OOD generalization. As shown in **Sec. 6**, our Jacobian model leverages locality and spatial symmetry of robot dynamics, enabling generalization to out-of-distribution robot motions.

Last but not least, our work suggests that for a robot dynamics learning method to be applicable to a wide range of robots, one should not always assume that sensors are *globally* precise. Our work grounds *local* visual measurements of the world to predict *local* Jacobian representations of the robotic system. Many robotic systems have imprecise global measurement through use or at creation. Many cable-driven soft robots produce cable readings that drift within hours, even though the morphology has not changed [29]. Our 3D-printed toy arm is built with highly affordable motors that experience backlash and report unreliable sensory measurements. Yet, as shown in our work, these systems are still useful for performing physical tasks if the dynamics model is strong enough to constantly perceive them in a closed loop. We hope this insight can encourage the robotics community to build a new generation of robotic hardware, given the increasingly capable robot modeling tools.

Visual Motion Representation for Robot Control. Our work rests on recent advancements in motion representations in computer vision [13, 51]. We use 2D motion supervision for training our Jacobian Fields and use point tracks for 2D robot control.

Past works have leveraged visual motion representations for robot control [57, 60, 59]. Ref. [57] uses points tracks to specify desired motion as inputs to the inverse differential kinematics controller. The paper proposes a data-mining technique that finds the nearest neighbor to track in the motion-tracking dataset. However, Ref. [57] relies on expert-based analytical Jacobians for the end-effector rigid movements. Constructing the analytical Jacobian is possible for rigid arm end-effector movements but not feasible for a broad range of robotic systems, as discussed in the related work section above. Ref. [59] fits a policy network that implicitly reconstructs a compressed representation of point tracks to provide detailed control guidance. While the work does not explicitly use the point track but a compressed form to guide learning, Ref. [59]'s results validate that point tracks are helpful for general imitation learning and could transfer between robot morphologies. Yet, Ref. [59] relies on an available kinematics and dynamics model for controlling the robot arm in the end-effector space. While Refs. [57, 59, 60] connect with our study in the space of visual motion representation for robot control, these works are motivated by and solve a different problem class. They cannot be directly applied to solve the modeling challenges presented in this paper but are prior validations of how visual motion representations can be used for robot control. In contrast, our work focuses on a set of multi-disciplinary challenges across fabrication, soft and bio-inspired robotics, and affordable automation.

12 Assumptions and Clarifications

12.1 Clarifications on Backlash

Backlash is commonly used in the robotics and kinematics communities to refer to a situation where a joint "wiggles", i.e., has play or slop, due to low-precision manufacturing, such as gears not fitting snugly. In this case, backlash leads to situations where joint sensors or proprioception of the robot report joint angles that are inaccurate and are not indicative of the true 3D configuration of the robot. Taking the DIY Poppy robot arm as an example, backlash causes the joint sensors to report the same values for different 3D configurations of the robot. Please see Figure 1 of Ref. [17] for more details. In this work, we showed that visual observations can resolve this issue by measuring the true extrinsic state of the system. When our approach is employed in closed-loop, as demonstrated in the results, we can command the Poppy DIY arm, a system experiencing backlash, to perform trajectory following accurately.

12.2 Details on Tracking Targets

We clarify that robotic manipulation tasks presented in this work have two types of tracking targets. For tasks involving the pneumatic hand standing up on a table (Fig. 3a, second row, main manuscript; Fig. 2(e, f), main manuscript), since the horizon is very short (around three control steps), we only have one final tracking target. For all other tasks, we have multiple tracking targets. We empirically create multiple tracking targets by storing a waypoint in the demonstration video every five to ten frames. We conduct the same procedure for both 2D and 3D trajectory tracking. Waypoints can also be specified manually if desired.

12.3 Assumptions on Second-order Transients

While our method can control a variety of robots, our method does not model second-order transients and imposes quasi-static assumptions. Our method assumes a quasi-static dynamical system, abstracting away second-order transients. While we agree that some manipulation tasks could require modeling second-order transients, our results in this work are consistent with prior work in manipulation planning. Ref. [35] has suggested that quasi-static models can hold up for a wide variety of manipulation tasks, including many that involve dexterous hands. These models only capture physical state transitions between equilibria. As Ref. [35] has theoretically and empirically shown, although modeling the transients allows the discovery of more dynamic behaviors in motion planning, the added computational complexity frequently outweighs the benefits. Our results align with prior works on these perspectives, as we have found that modeling transitions between static equilibria can enable control of diverse and complex robotic systems. Unlike prior works that assume a physical description of the system is provided by an expert, our work shows that the linearization between steady states can be used to learn volumetric Jacobians via visual observations.

References

[1] Allegro hand, wonik robotics. URL http://wonikrobotics.com/robot-hand. Accessed: 2024-06-17.

[2] Assemble poppy ergo jr, documentation of the poppy platform, . URL https://docs.poppy-project.org/en/assembly-guides/ergo-jr/index.html. Accessed: 2024-06-17.

[3] Poppy project - ergo jr, . URL https://www.poppy-project.org/en/robots/poppy-ergo-jr/. Accessed: 2024-06-17.

[4] P. W. Battaglia, R. Pascanu, M. Lai, D. Rezende, and K. Kavukcuoglu. Interaction networks for learning about objects, relations and physics, 2016. URL https://arxiv.org/abs/1612.00222.

[5] J. Bonet and R. D. Wood. *Nonlinear continuum mechanics for finite element analysis*. Cambridge university press, Cambridge, UK, 1997.

[6] R. S. Bowen, R. Tucker, R. Zabih, and N. Snavely. Dimensions of motion: Monocular prediction through flow subspaces. In *2022 International Conference on 3D Vision (3DV)*, pages 454–464. IEEE, 2022.

[7] T. J. Buchner, S. Rogler, S. Weirich, Y. Armati, B. G. Cangan, J. Ramos, S. T. Twiddy, D. M. Marini, A. Weber, D. Chen, et al. Vision-controlled jetting for composite systems and robots. *Nature*, 623(7987):522–530, 2023.

[8] M. Caron, H. Touvron, I. Misra, H. Jégou, J. Mairal, P. Bojanowski, and A. Joulin. Emerging properties in self-supervised vision transformers, 2021. URL https://arxiv.org/abs/2104.14294.

[9] B. Chen, R. Kwiatkowski, C. Vondrick, and H. Lipson. Fully body visual self-modeling of robot morphologies. *Science Robotics*, 7(68):eabn1944, 2022.

[10] M. Chevalier-Boisvert, G. Alain, F. Golemo, and D. Nowrouzezahrai. Robo-planet: Learning to poke in a day. *arXiv preprint arXiv:1911.03594*, 2019.

[11] C. Chi, S. Feng, Y. Du, Z. Xu, E. Cousineau, B. Burchfiel, and S. Song. Diffusion policy: Visuomotor policy learning via action diffusion. In *Proceedings of Robotics: Science and Systems (RSS)*, 2023.

[12] M. Cuturi. Sinkhorn distances: Lightspeed computation of optimal transportation distances, 2013. URL https://arxiv.org/abs/1306.0895.

[13] C. Doersch, Y. Yang, M. Vecerik, D. Gokay, A. Gupta, Y. Aytar, J. Carreira, and A. Zisserman. Tapir: Tracking any point with per-frame initialization and temporal refinement. In *Proceedings of the IEEE/CVF International Conference on Computer Vision*, pages 10061–10072, 2023.

[14] J. Feydy, T. Séjourné, F.-X. Vialard, S.-i. Amari, A. Trouvé, and G. Peyré. Interpolating between optimal transport and mmd using sinkhorn divergences. In *The 22nd International Conference on Artificial Intelligence and Statistics*, pages 2681–2690. PMLR, 2019.

[15] P. R. Florence, L. Manuelli, and R. Tedrake. Dense object nets: Learning dense visual object descriptors by and for robotic manipulation. *arXiv preprint arXiv:1806.08756*, 2018.

[16] S. Funabashi, T. Isobe, S. Ogasa, T. Ogata, A. Schmitz, T. P. Tomo, and S. Sugano. Stable in-grasp manipulation with a low-cost robot hand by using 3-axis tactile sensors with a cnn. In *2020 IEEE/RSJ International Conference on Intelligent Robots and Systems (IROS)*, pages 9166–9173. IEEE, 2020.

[17] F. Golemo, A. A. Taiga, A. Courville, and P.-Y. Oudeyer. Sim-to-real transfer with neural-augmented robot simulation. In *Conference on Robot Learning*, pages 817–828. PMLR, 2018.

[18] C. Hegde, J. Su, J. M. R. Tan, K. He, X. Chen, and S. Magdassi. Sensing in soft robotics. *ACS nano*, 17(16): 15277–15307, 2023.

[19] Y. Hong, K. Zhang, J. Gu, S. Bi, Y. Zhou, D. Liu, F. Liu, K. Sunkavalli, T. Bui, and H. Tan. Lrm: Large reconstruction model for single image to 3d, 2023.

[20] F. Ilievski, A. D. Mazzeo, R. F. Shepherd, X. Chen, and G. M. Whitesides. Soft robotics for chemists. *Angewandte Chemie International Editiion*, 2011.

[21] A. Kirillov, E. Mintun, N. Ravi, H. Mao, C. Rolland, L. Gustafson, T. Xiao, S. Whitehead, A. C. Berg, W.-Y. Lo, et al. Segment anything. In *Proceedings of the IEEE/CVF International Conference on Computer Vision*, pages 4015–4026, 2023.

[22] D. Koschier, J. Bender, B. Solenthaler, and M. Teschner. Smoothed particle hydrodynamics techniques for the physics based simulation of fluids and solids. *arXiv preprint arXiv:2009.06944*, 2020.

[23] W. J. Krzanowski. *Principles of multivariate analysis: a user's perspective*. Oxford University Press, Inc., USA, 1988. ISBN 0198522118.

[24] M. Lambeta, P.-W. Chou, S. Tian, B. Yang, B. Maloon, V. R. Most, D. Stroud, R. Santos, A. Byagowi, G. Kammerer, et al. Digit: A novel design for a low-cost compact high-resolution tactile sensor with application to in-hand manipulation. *IEEE Robotics and Automation Letters*, 5(3):3838–3845, 2020.

[25] M. Lapeyre. *Poppy: open-source, 3D printed and fully-modular robotic platform for science, art and education*. PhD thesis, Université de Bordeaux, 2015.

[26] Y. Li, J. Wu, R. Tedrake, J. B. Tenenbaum, and A. Torralba. Learning particle dynamics for manipulating rigid bodies, deformable objects, and fluids, 2019. URL `https://arxiv.org/abs/1810.01566`.

[27] T.-Y. Lin, M. Maire, S. Belongie, J. Hays, P. Perona, D. Ramanan, P. Dollár, and C. L. Zitnick. Microsoft coco: Common objects in context. In *Computer Vision–ECCV 2014: 13th European Conference, Zurich, Switzerland, September 6-12, 2014, Proceedings, Part V 13*, pages 740–755. Springer, 2014.

[28] J. I. Lipton, R. MacCurdy, Z. Manchester, L. Chin, D. Cellucci, and D. Rus. Handedness in shearing auxetics creates rigid and compliant structures. *Science*, 360(6389):632–635, 2018.

[29] C. Liu, A. Moncada, H. Matusik, D. I. Erus, and D. Rus. A modular bio-inspired robotic hand with high sensitivity. In *2023 IEEE International Conference on Soft Robotics (RoboSoft)*, pages 1–7. IEEE, 2023.

[30] K. Lynch. *Modern Robotics*. Cambridge University Press, Cambridge, UK, 2017.

[31] C. Majidi. Soft-matter engineering for soft robotics. *Advanced Materials Technologies*, 4(2):1800477, 2019.

[32] H. Matusik, C. Liu, and D. Rus. Directly 3d printed, pneumatically actuated multi-material robotic hand. *arXiv preprint arXiv:2310.16280*, 2023.

[33] L. Mescheder, M. Oechsle, M. Niemeyer, S. Nowozin, and A. Geiger. Occupancy networks: Learning 3d reconstruction in function space. In *Proceedings of the IEEE/CVF conference on computer vision and pattern recognition*, pages 4460–4470, 2019.

[34] B. Mildenhall, P. P. Srinivasan, M. Tancik, J. T. Barron, R. Ramamoorthi, and R. Ng. NeRF: Representing scenes as neural radiance fields for view synthesis. In *Proceedings of the European Conference on Computer Vision (ECCV)*, pages 405–421, 2020.

[35] T. Pang, H. J. T. Suh, L. Yang, and R. Tedrake. Global planning for contact-rich manipulation via local smoothing of quasi-dynamic contact models, 2023. URL `https://arxiv.org/abs/2206.10787`.

[36] S. Peng, M. Niemeyer, L. M. Mescheder, M. Pollefeys, and A. Geiger. Convolutional occupancy networks. *CoRR*, abs/2003.04618, 2020. URL `https://arxiv.org/abs/2003.04618`.

[37] X. B. Peng, M. Andrychowicz, W. Zaremba, and P. Abbeel. Sim-to-real transfer of robotic control with dynamics randomization. In *2018 IEEE international conference on robotics and automation (ICRA)*, pages 3803–3810. IEEE, 2018.

[38] T. Pfaff, M. Fortunato, A. Sanchez-Gonzalez, and P. W. Battaglia. Learning mesh-based simulation with graph networks, 2021. URL `https://arxiv.org/abs/2010.03409`.

[39] F. Renda, V. Cacucciolo, J. Dias, and L. Seneviratne. Discrete cosserat approach for soft robot dynamics: A new piece-wise constant strain model with torsion and shears. In *2016 IEEE/RSJ International Conference on Intelligent Robots and Systems (IROS)*, pages 5495–5502. IEEE, 2016.

[40] D. Rus and M. T. Tolley. Design, fabrication and control of soft robots. *Nature*, 521(7553):467–475, 2015.

[41] E. Salvato, G. Fenu, E. Medvet, and F. A. Pellegrino. Characterization of modeling errors affecting performances of a robotics deep reinforcement learning controller in a sim-to-real transfer. In *2021 44th International Convention on Information, Communication and Electronic Technology (MIPRO)*, pages 1154–1159. IEEE, 2021.

[42] A. Sanchez-Gonzalez, J. Godwin, T. Pfaff, R. Ying, J. Leskovec, and P. W. Battaglia. Learning to simulate complex physics with graph networks, 2020. URL `https://arxiv.org/abs/2002.09405`.

[43] K. Shaw, A. Agarwal, and D. Pathak. Leap hand: Low-cost, efficient, and anthropomorphic hand for robot learning. *arXiv preprint arXiv:2309.06440*, 2023.

[44] B. Shen, Z. Jiang, C. Choy, L. J. Guibas, S. Savarese, A. Anandkumar, and Y. Zhu. Acid: Action-conditional implicit visual dynamics for deformable object manipulation, 2022. URL `https://arxiv.org/abs/2203.06856`.

[45] H. Shi, H. Xu, Z. Huang, Y. Li, and J. Wu. Robocraft: Learning to see, simulate, and shape elasto-plastic objects with graph networks, 2022. URL `https://arxiv.org/abs/2205.02909`.

[46] J. Shintake, V. Cacucciolo, D. Floreano, and H. Shea. Soft robotic grippers. *Advanced materials*, 30(29): 1707035, 2018.

[47] E. Sifakis and J. Barbic. Fem simulation of 3d deformable solids: a practitioner's guide to theory, discretization and model reduction. In *ACM SIGGRAPH 2012 Courses*, SIGGRAPH '12, New York, NY, USA, 2012. Association for Computing Machinery. ISBN 9781450316781. doi: 10.1145/2343483.2343501. URL `https://doi.org/10.1145/2343483.2343501`.

[48] M. Stölzle, D. Rus, and C. Della Santina. An experimental study of model-based control for planar handed shearing auxetics robots. In *International Symposium on Experimental Robotics*, pages 153–167. Springer, 2023.

[49] J. Tang, Z. Chen, X. Chen, T. Wang, G. Zeng, and Z. Liu. Lgm: Large multi-view gaussian model for high-resolution 3d content creation, 2024. URL `https://arxiv.org/abs/2402.05054`.

[50] R. Tedrake and the Drake Development Team. Drake: Model-based design and verification for robotics, 2019. URL `https://drake.mit.edu`.

[51] Z. Teed and J. Deng. RAFT: Recurrent all-pairs field transforms for optical flow. In *Proceedings of the European Conference on Computer Vision (ECCV)*, 2020.

[52] A. Tewari, T. Yin, G. Cazenavette, S. Rezchikov, J. B. Tenenbaum, F. Durand, W. T. Freeman, and V. Sitzmann. Diffusion with forward models: Solving stochastic inverse problems without direct supervision. *Advances in Neural Information Processing Systems (NeurIPS)*, 2023.

[53] J. Tobin, R. Fong, A. Ray, J. Schneider, W. Zaremba, and P. Abbeel. Domain randomization for transferring deep neural networks from simulation to the real world. In *2017 IEEE/RSJ international conference on intelligent robots and systems (IROS)*, pages 23–30. IEEE, 2017.

[54] D. Trivedi, C. D. Rahn, W. M. Kier, and I. D. Walker. Soft robotics: Biological inspiration, state of the art, and future research. *Applied bionics and biomechanics*, 5(3):99–117, 2008.

[55] R. L. Truby, L. Chin, and D. Rus. A recipe for electrically-driven soft robots via 3d printed handed shearing auxetics. *IEEE Robotics and Automation Letters*, 6(2):795–802, 2021.

[56] R. L. Truby, L. Chin, A. Zhang, and D. Rus. Fluidic innervation sensorizes structures from a single build material. *Science advances*, 8(31):eabq4385, 2022.

[57] M. Vecerik, T. Hester, J. Scholz, F. Wang, O. Pietquin, B. Piot, N. Heess, T. Rothörl, T. Lampe, and M. Riedmiller. Leveraging demonstrations for deep reinforcement learning on robotics problems with sparse rewards, 2018. URL `https://arxiv.org/abs/1707.08817`.

[58] H. Wang, M. Totaro, and L. Beccai. Toward perceptive soft robots: Progress and challenges. *Advanced Science*, 5(9):1800541, 2018.

[59] C. Wen, X. Lin, J. So, K. Chen, Q. Dou, Y. Gao, and P. Abbeel. Any-point trajectory modeling for policy learning, 2024. URL `https://arxiv.org/abs/2401.00025`.

[60] Z. Xu, Z. He, J. Wu, and S. Song. Learning 3d dynamic scene representations for robot manipulation, 2020. URL `https://arxiv.org/abs/2011.01968`.

[61] A. Yu, V. Ye, M. Tancik, and A. Kanazawa. pixelNeRF: Neural radiance fields from one or few images. In *Proceedings of the IEEE Conference on Computer Vision and Pattern Recognition (CVPR)*, 2021.

[62] A. Zhang, R. L. Truby, L. Chin, S. Li, and D. Rus. Vision-based sensing for electrically-driven soft actuators. *IEEE Robotics and Automation Letters*, 7(4):11509–11516, 2022.

[63] A. Zhang, T.-H. Wang, R. L. Truby, L. Chin, and D. Rus. Machine learning best practices for soft robot proprioception. In *2023 IEEE/RSJ International Conference on Intelligent Robots and Systems (IROS)*, pages 2564–2571. IEEE, 2023.

[64] H. Zhu, A. Gupta, A. Rajeswaran, S. Levine, and V. Kumar. Dexterous manipulation with deep reinforcement learning: Efficient, general, and low-cost. In *2019 International Conference on Robotics and Automation (ICRA)*, pages 3651–3657. IEEE, 2019.
